# Predicting the success of induction of labour using cervical volume

**DOI:** 10.1186/s13104-021-05865-5

**Published:** 2021-12-18

**Authors:** Malitha Patabendige, Sanka Rajesh Athulathmudali

**Affiliations:** 1 Base Hospital, Pottuvil, Sri Lanka; 2 Werribee Mercy Hospital, Melbourne, VIC Australia

**Keywords:** Induction of labour, Bishop Score, Cervical volume, 2D Ultrasound, Successful induction

## Abstract

**Objectives:**

Assessing the likelihood of success of induction of labour using cervical volume is an important research question.

**Data description:**

We provide data generated in a prospective observational study which was carried out at North Colombo Teaching Hospital, Ragama, Sri Lanka. Study conducted to compare pre-induction digital cervical assessment, sonographic cervical length, and sonographic cervical volume with vaginal delivery rate within 24 h. Inductions with 100 singleton pregnancies at term were included.

## Objectives

Induction of labour is one of the most commonly applying obstetric interventions in the contemporary obstetric practice [1]. Vaginal progesterone E_2_ and transcervical Foley catheter are the common methods of induction of labour practicing in Sri Lanka [2–4]. Sonographic cervical volume can be computed using the length and the diameter of the cervix assuming a cylinder macroscopically. This might have the possibility to cover two cervical scoring systems [cervical length and Bishop score] combined in a single measurement. Objectives were to describe the association between cervical volume, cervical length and Bishop score and the vaginal delivery rate within 24 h. The findings based on these data have been published in 2021 [5].

## Data description

### Study design and setting

The provided data were gathered for a prospective observational study which was carried out at North Colombo Teaching Hospital (NCTH), Ragama, Sri Lanka. Eligible pregnant women were selected after hospital admission for the elective induction of labour.

### Patient selection process

All the consecutive inductions performed over a two-month period were assessed for eligibility and eligible women were invited to participate. Informed written consent was obtained prior to data collection. Singleton pregnancies with vertex presentation at term with normal body mass index at the booking antenatal visit and those with an unfavourable cervix (Bishop score < 6) at the time of hospital admission were included. These women were induced electively after 37 weeks of gestation for various obstetric indications and majority were after 40 weeks (past delivery date). Other obstetric indications included in the study were pregnancy-induced hypertension without evidence of fetal compromise, and well-controlled gestational diabetes mellitus with adequate fetal growth. Those with previous caesarean delivery, myomectomy, multiple pregnancies, malpresentation, pre-labour rupture of membranes, evidence of fetal compromise, history of previous cervical surgeries, Bishop score more than 6 at the time of hospital admission and women with established labour were excluded from the study.

### Protocol of induction of labour

Local protocol for the induction of labour has prepared according to the National Institute of Clinical Excellence (NICE) clinical guideline for induction of labour and the guideline prepared by the Sri Lanka College of Obstetricians Gynaecologists [4–6]. Further details have mentioned in the original article with the results [5].

### Study procedure and data collection

After admission, the pre-induction cervical assessment was performed using both the Bishop score and transvaginal ultrasound scan (TVUS). Bishop score was calculated after vaginal examination by the treating physician in the ward. Thereafter, TVUS was conducted by the investigator (SRA) for cervical volume and cervical length followed by the induction of labour. The procedure for TVUS was standardised according to the recommendations for cervical length assessment published by the International Society of Ultrasound in Obstetrics and Gynecology (ISUOG) [7]. TVUS was performed with a sector phased array of 7 MHz probes (Samsung Medison Co. Ltd-Korea), according to the ISUOG practice advice [7]. Three measurements were taken and the shortest measurement was taken as the final cervical length. Measurement of the anteroposterior diameter of the cervix was obtained at the midpoint of the cervix, right-angled to the endocervical canal. Cervical volume was calculated assuming the cervix as a cylinder in geometric view (V = πr^2^h). Figure in the data file 2 shows calliper placement of cervical length (a) and mid-cervical diameter (b) when visualized through TVUS [8].

### Outcome measures

Total of 100 pregnant women were studied. Outcome measures were independent variables (cervical volume, cervical length, and Bishop score), dependent variables (vaginal delivery rate within 24 h and induction to delivery interval). Data have been entered in a SPSS Spreadsheet and included in Table 1 along with a read me file [9, 10]. Ethical approval was obtained from the Ethics Review Committee, Faculty of Medicine, University of Kelaniya, Sri Lanka. This study was one of the earlier works on the cervical volume in induction of labour.

**Table 1:** Overview of data files

Label	Name of data file/data set	File types (file extension)	Data repository and identifier (DOI)
Data file 1	SPSS Cervical volume BMC RSN Datasheet Nov 2021	SPSS (.sav)	Figshare [9] (https://doi.org/10.6084/m9.figshare.15034956.v2)
Data file 2	Fig 1	JPEG (.jpg)	Figshare [8] (https://doi.org/10.6084/m9.figshare.17041505.v1)
Data file 3	Read me file for cervical volume SPSS	TEXT (.txt)	Figshare [10] (https://doi.org/10.6084/m9.figshare.17086436.v1)

## Limitations

- Data collection was done using a convenient sampling method.
- The results may not be generalizable since the study was carried out in a single center.
- Small sample size.
- Analysis would have been better if there were several sonographers to measure the cervical volume giving a chance to compute inter-observer variability.
- As this study is one of the preliminary works on the cervical volume, reusability of the data might be limited until further studies are available.
- This might not be a practical, reproducible, simple measurement and might not yield any useful data to the day to day obstetrician to rely on unless strong evidence proves its success.

## Data Availability

The data described in this Data note can be freely and openly accessed on [https://doi.org/10.6084/m9.figshare.15034956.v2]. Please see Table 1 and reference list for details and links to the data [9]. Table has included the link to the figure as data file 2 [8] [https://doi.org/10.6084/m9.figshare.17041505.v1].

## Abbreviations

NCTH: North Colombo Teaching Hospital
NICE: National Institute of Clinical Excellence
ISUOG: International Society of Ultrasound in Obstetrics and Gynecology
TVUS: Transvaginal ultrasound
